# The Oncofetal Protein IMP3: A Novel Grading Tool and Predictor of Poor Clinical Outcome in Human Gliomas

**DOI:** 10.1155/2015/413897

**Published:** 2015-01-28

**Authors:** Alessandro Del Gobbo, Valentina Vaira, Lucia Ferrari, Carlo Patriarca, Andrea Di Cristofori, Dario Ricca, Manuela Caroli, Paolo Rampini, Silvano Bosari, Stefano Ferrero

**Affiliations:** ^1^Division of Pathology, Fondazione IRCCS Ca' Granda-Ospedale Maggiore Policlinico, 20122 Milan, Italy; ^2^Division of Pathology, Ospedale Sant'Anna, 22020 Como, Italy; ^3^Division of Neurosurgery, Fondazione IRCCS Ca' Granda-Ospedale Maggiore Policlinico, 20122 Milan, Italy; ^4^Department of Pathophysiology and Organ Transplant, University of Milan, 20122 Milan, Italy; ^5^Department of Biomedical, Surgical and Dental Sciences, University of Milan, 20122 Milan, Italy

## Abstract

Morphologic criteria illustrated in WHO guidelines are the most significant prognostic factor in human gliomas, but novel biomarkers are needed to identify patients with a poorer outcome. The present study examined the expression of the oncofetal protein IMP3 in a series of 135 patients affected by high-grade (grade III and IV) gliomas, correlating the results with proliferative activity, molecular parameters, and clinical and follow-up data. Overall, IMP3 expression was higher in glioblastomas (68%) than in grade III tumors (20%, *P* < 0.0001), and IMP3-positive high-grade gliomas showed a shorter overall and disease-free survival than negative ones (*P* = 0.0002 and *P* = 0.006, resp.). IMP3 expression was significantly associated with the absence of mutations of IDH1 gene (*P* = 0.0001) and with the unmethylated phenotype of MGMT in high-grade gliomas (*P* = 0.004). High Ki67 levels were correlated with better prognosis in glioblastomas but IMP3 expression was not correlated with the proliferation index. These findings confirm the role of IMP3 as a marker of poor outcome, also in consideration of its association with IDH1 wild-type phenotype and MGMT unmethylated status. The data suggest that IMP3 staining could identify a subgroup of patients with poor prognosis and at risk of recurrence in high-grade gliomas.

## 1. Introduction

The insulin-like growth factor II mRNA-binding protein family comprises three proteins (IMP1, IMP2, and IMP3) that regulate mRNA transport, translation, and turnover and their function is implicated in cell proliferation, adhesion, invasion, and migration [1].

Their expression is almost exclusively restricted to the early stages of embryogenesis, and, in particular, IMP3 is epigenetically silenced soon after birth, with little or no detectable protein in normal adult tissues [2].

Its reexpression is observed in a wide spectrum of human tumors, including ovarian, serous endometrial, and cervical adenocarcinomas, as well as lung, pleural, gastric, colorectal, renal, and bladder cancers and in lung tumors with neuroendocrine differentiation, but not in benign counterparts [3, 4].

In addition, IMP3 reexpression is correlated with tumor aggressiveness and unfavorable prognosis and it is also considered a marker of preinvasive lesions [5]. We recently identified IMP3 as a biomarker for distinguishing atypical premalignant endometriotic cysts from endometriotic cyst with reactive changes [6].

Gliomas are the most common primary brain cancers in adults, characterized, especially for higher grades, by poor prognosis. The World Health Organization (WHO) identifies three major glioma histotypes (astrocytoma, oligodendroglioma, and mixed oligoastrocytoma) graded according to the neoplastic cellular density, number of mitotic figures, presence or absence of nuclear atypia, neovascularization, and necrosis. This grading system, based exclusively on morphology, is the most significant prognostic factor in predicting glioma patients' survival [7]. Moreover, even if it is not included as a grading parameter in the current WHO classification, the proliferative index, measured by Ki67 immunoreactivity, is commonly employed by pathologists to support the diagnosis and to grade glioma [8, 9].

Molecular characteristics, including methylation of O6-methylguanine-DNA methyltransferase (MGMT) promoter, isocitrate dehydrogenase 1 or 2 (IDH1/2) gene mutations, or 1p/19q chromosomal loci deletion, are currently used to genetically classify glioma patients. Therapeutical strategies commonly employed by neurooncologists include surgery and radiotherapy but prognosis remains poor especially for adult patients and for high-grade tumors [10–13]. Therefore, novel biomarkers are needed to improve accuracy of histological diagnosis and to provide more accurate prognostic information [14, 15].

Previous studies identified IMP3 as a glioblastoma-specific marker and as a prognostic factor in series of 83 glioblastomas and in a series of 77 pediatric pilocytic and pilomyxoid astrocytomas, though only in the latter confirmed by multivariate analysis [16, 17].

Since the IMP3 expression in tumoral tissues has been correlated with decreased survival and increased risk of progression and metastases and Ki67 index is a parameter that is helpful in grading gliomas, in this study we evaluated IMP3 expression and Ki67 labeling index in a series of adult patients with high-grade gliomas.

The aim of this study was to evaluate clinical significance of the immunohistochemical expression of IMP3 in high-grade gliomas and to investigate its role as a diagnostic and prognostic biomarker.

## 2. Materials and Methods

### 2.1. Patients

We enrolled a series of 165 patients diagnosed with glioma that consecutively underwent surgery for therapeutic purposes at Fondazione IRCCS Ca' Granda Hospital (Milan, Italy) between 2008 and 2011. The Hospital Institutional Review Board approved the study. Gliomas were diagnosed following the WHO classification. According to these criteria, 10 and 19 patients were diagnosed as anaplastic oligodendrogliomas (AO) or astrocytomas (AA), respectively, (grade III) and 106 as glioblastomas (GBM, grade IV).

Patients' clinicopathological characteristics are detailed in Table 1.

All the patients were treated with surgical resection of the lesion. Extent of resection was evaluated on brain MRI performed within 3 days after surgery. Gross total resection (GTR) was defined as more than 95% of tumor volume removed and could be obtained in fifteen (52%) grade III glioma and in 70 (66%) GBM patients. Subtotal resection (STR, corresponding to removal of 80–95% of the tumor) and partial resection (PR, removal of less than 80% of the tumor) were achieved in 6 (20%) and 8 (28%) grade III patients, respectively. Nineteen (18%) GBM patients received a STR and 8 (7,5%) a PR. MR imaging scans were read blinded by neuroradiologists and surgeons (Manuela Caroli, Andrea Di Cristofori, and Paolo Rampini) and postoperative scans (contrast enhancement) were analyzed to determine the extent of the resection. GBM patients had a median Karnofsky performance score (KPS) at diagnosis of 75 (range: 50–100). Following histological diagnosis, all patients underwent concomitant chemoradiation therapy according to Stupp's protocol.

Follow-up of patients consisted of neurooncological assessment and brain MRI with gadolinium every 3 months. Disease-free survival (DFS) was calculated from surgery to tumor progression determined by radiological (brain MRIs) procedures, whereas overall survival (OS) was calculated from surgery to patients' death. Follow-up ended in December 2012.

### 2.2. Molecular Characterization of Gliomas

Promoter methylation status of the O(6)-methylguanine-DNA methyltransferase (MGMT) gene was analyzed in all cases using the CpGenome DNA Modification Kit followed by CpG-WIZ MGMT-Methylation specific PCR assay (Millipore Corporation, Billerica, MA, USA) following manufacturer instruction.

### 2.3. Immunohistochemistry

Routinely prepared formalin-fixed paraffin-embedded blocks were used to construct 6 paraffin-embedded tissue microarrays, as previously described, with slight modifications [18]. Briefly, for this series, we sampled one core of normal brain tissue and four cores of tumor tissue for each case.

One mm diameter cores were generated using a semiautomatic arrayer (Alphelys Minicore2, Plaisir, France) and each tissue microarray block contained up to 165 cores with a total of 980 spots, with 5 spots per case.

Before immunohistochemistry (IHC), analyses were performed; a section from each tissue microarray block was cut and stained with hematoxylin and eosin for morphological evaluation.

All cells of each tissue core included in the TMAs were evaluated, and only cases containing two or more preserved tissue cores were scored.

IDH1 c.395G>A mutation was investigated using IDH1^R132H^ mouse monoclonal antibody (clone H09, Dianova GmbH, Hamburg, Germany) that has been demonstrated to be a reliable method for evaluation of this mutation status with no false negative cases [19, 20].

IMP3 and Ki67 immunohistochemical expression were evaluated by using IMP3 antibody (M3626), a mouse monoclonal antibody specific for IMP3/KOC antigen (clone 69.1, DAKO, Carpenteria, CA, USA), and Ki67 antibody (MIB1) by DAKO.

Immunohistochemistry was performed using the automatic system BenchMark XT (Ventana Medical Systems, Inc., Tucson, AZ, USA). Reactions were revealed using the UltraViewTM Universal DAB, a biotin-free, multimer-based detection system, according to the manufacture's instruction.

Sections of pancreatic carcinoma known to express IMP3 were used as positive controls.

For negative and positive controls, we replaced the primary antibody with nonimmune IgG or we incubated a human placenta with IMP3 antibody, respectively.

Positive staining for IMP3 was defined as a high intensity, dark brown cytoplasmic staining in at least 10% of tumor cells of the tissue microarray cores easily observed at low-power magnification (10x) and was scored as focal (≤30%) or diffuse (>30%). The scores from each core stained with IMP3 in the same patient were averaged to obtain a mean value.

The percentage of Ki67-positive cells (Ki67 labeling index) was scored in tumor hot-spots from four high-power fields on full section slides. The highest Ki67 percentage in each case was then recorded and used as proliferation index.

Immunohistochemical slides from all the cases were blind-reviewed by three expert pathologists (Alessandro Del Gobbo, Stefano Ferrero, and Lucia Ferrari). When discrepancies occurred, the three pathologists reviewed the case to find an agreement score.

### 2.4. Statistical Analysis

Groups' comparisons were performed using univariate two-sided Student's *t*-test or Mann-Whitney *U* test when appropriate. The significance of a variable for patients' prognosis was analyzed using the Cox regression hazard model as either univariate or multivariate analysis (MedCalc Software, Mariakerke, Belgium) considering proteins IHC score, MGMT methylation status, or tumor grade as categorical variables. For Ki67 immunoreactivity scores, cut-offs to separate patients into low- or high-expressor groups were generated using receiver operating characteristics (ROC) curves using the nonarbitrary criterion derived from the Youden index (*J*, MedCalc Software). The *J* index is defined as sensitivity plus specificity minus 1. We calculated the *J* index on the merged series of patients for which follow-up data were available (*n* = 94) and then we separately applied it as cut-off on the grade III or grade IV sets to consistently test the predictive effect of the variable on different patients' series. The Kaplan-Meier method was used to plot survival curves when patients were categorized into two groups based on the variable. In disease-free survival analysis, patients' death was censored. Difference in survival curves was computed using the log-rank test or by Cox's proportional-hazards regression model (MedCalc Software). Two-sided *P* values less than 0.05 were considered statistically significant.

## 3. Results

IMP3 expression could be evaluated in all 165 patients affected by primary gliomas. All patients were treated with surgical removal and nobody received a diagnostic-only procedure. Disease-free survival data were available for 14 grade III (49%) and 64 GBM patients (60%), respectively. Conversely, overall survival data were available for 22 grade III gliomas (76%) and 74 GBMs (69%). IDH1 and MGMT immunohistochemical and molecular profile were evaluated in all grade III tumors and in 95 (90%) GMBs, respectively.

**Figure 1:**
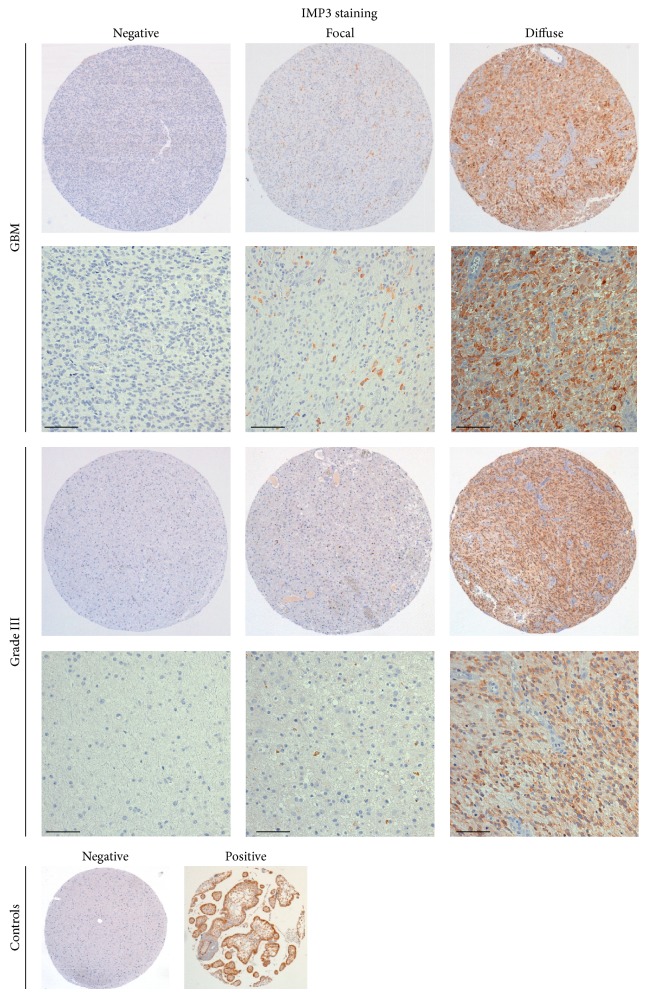
IMP3 staining in HGGs. Representative images of negative, focally, or diffuse positive cytoplasmic staining are shown for GBM or grade III glioma together with negative and positive (human placenta) controls. Original magnification for TMA spot is 40x. Scale bar indicates 100 *μ*m.

Overall, IMP3 expression was found in 74 GBMs (68%) and 6 grade III tumors (20%, *P* < 0.0001). With regard to grade III tumors, 5 out of 6 (84%) IMP3-positive gliomas showed an astrocytic differentiation (Table 2). GBMs showed a diffuse pattern of staining in 42 out of 74 positive cases (58%; Figure 1), whereas grade III tumors displayed this staining pattern in 2 out of 6 positive cases (33%; Figure 1).

**Figure 2:**
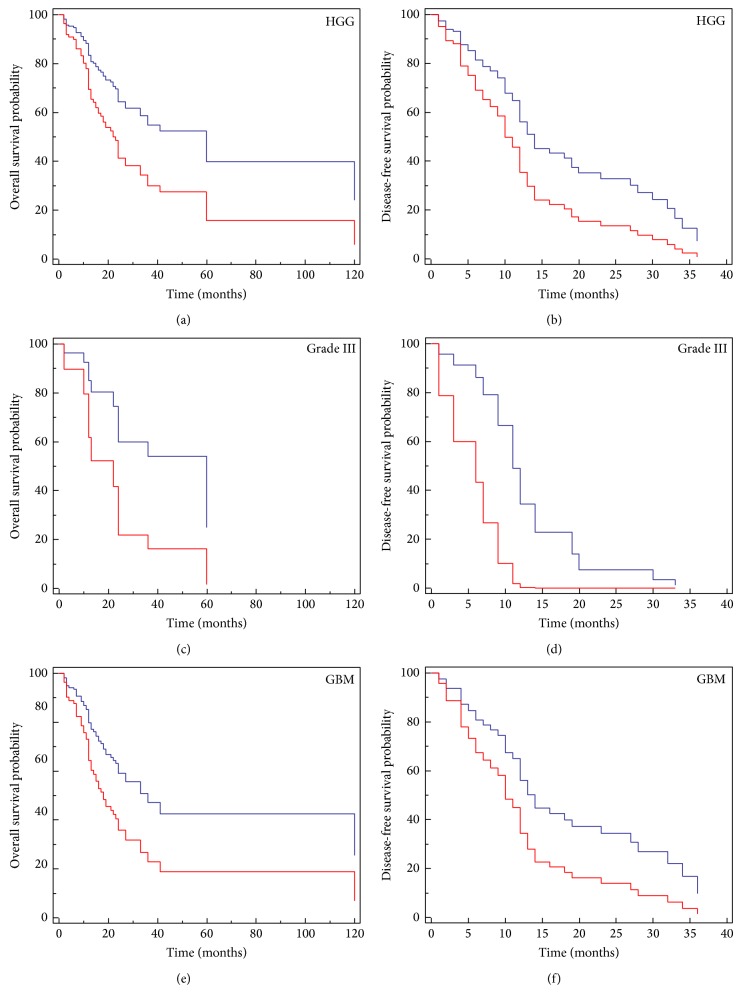
Cox proportional-hazards regression analyses of IMP3-positive and IMP3-negative HGGs for overall ((a), (c), and (e)) or disease-free survival ((b), (d), and (f)). IMP3 presence is a poor prognostic factor either considering high-grade gliomas (*P* = 0.0002, HR: 0.3392, 95% CI: 0.1928–0.5965 and *P* = 0.006, HR: 0.4586, 95% CI: 0.2631 to 0.7994 for OS and DFS, resp.) or separately grade III (*P* = 0.02, HR: 4.47, 95% CI: 1.19–16.8 and *P* = 0.02, HR: 13.30, 95% CI: 1.44–122.6 for OS and DFS, resp.) and GBM (*P* = 0.01, HR: 0.41, 95% CI: 0.19 to 0.84 and *P* = 0.04, HR: 0.5, 95% CI: 0.23–0.98 for OS and DFS, resp.) patients.

We next investigated whether IMP3 expression could be a prognostic marker for high-grade gliomas, and we found that immunoreactive IMP3-HGG showed a shorter overall and disease-free survival than IMP3-negative cases (*P* = 0.0002 and *P* = 0.006, resp.; Figure 2(a)). This result was confirmed analysing separately grade III (Figures 2(c) and 2(d)) or GBM (Figures 2(e) and 2(f)) patients for disease-free or overall survival times.

No difference was found when correlating focal or diffuse IMP3 staining with disease-free and overall survival, and no statistically significant correlations were observed with the other demographic or clinicopatholagical parameters such as age or sex.

**Figure 3:**
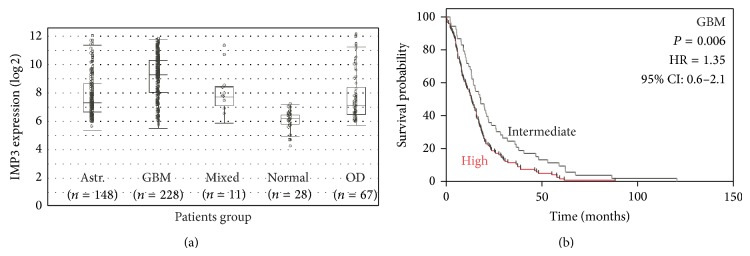
*In silico* analysis of IMP3 levels in human gliomas. The Rembrandt database was queried for IMP3 gene (IGF2BP3) expression in gliomas and in nonneoplastic counterparts (a) or for correlation of IMP3 overexpression with GBM patients' prognosis (*n* = 167 GBM patients) (b). Kaplan-Meier survival curves were generated for GBM with unchanged IMP3 expression (fold-change 0.8–1.2, intermediate group, *n* = 44) or overexpression (fold-change ≥5, high group, *n* = 123). *P* values are from log-rank test. Astr.: astrocytoma; OD: oligodendroglioma; normal: nonneoplastic brain parenchyma; GBM: glioblastoma.

Analysis of public database such as Oncomine (https://www.oncomine.org/resource/login.html) or Rembrandt (http://rembrandt.nci.nih.gov) for IMP3 (gene ID: IGF2BP3) gene expression or gene amplification in human gliomas showed that it was overexpressed by HGGs and specifically by GBM (Table 3 and Figure 3(a)) and also correlated with poor prognosis (Figure 3(b)), thus confirming our protein expression data.

With regard to molecular characteristics, mutation of IDH1 gene (R132H) as detected by immunohistochemistry was more frequently present in grade III gliomas than in GBMs (*P* < 0.0001 by Chi-square), and in GBMs its presence was a favorable prognostic marker being associated with a higher DFS and OS (*P* < 0.0001 and *P* = 0.0001, resp.).

Methylation of MGMT gene promoter was more frequently found in GBMs than in grade III gliomas (*P* < 0.0017 by Chi-square) and it was significantly associated with better OS and DFS in GBM (*P* < 0.0001 and *P* = 0.0001, resp.), whereas it was not associated with other demographic parameters such as age or sex.

**Figure 4:**
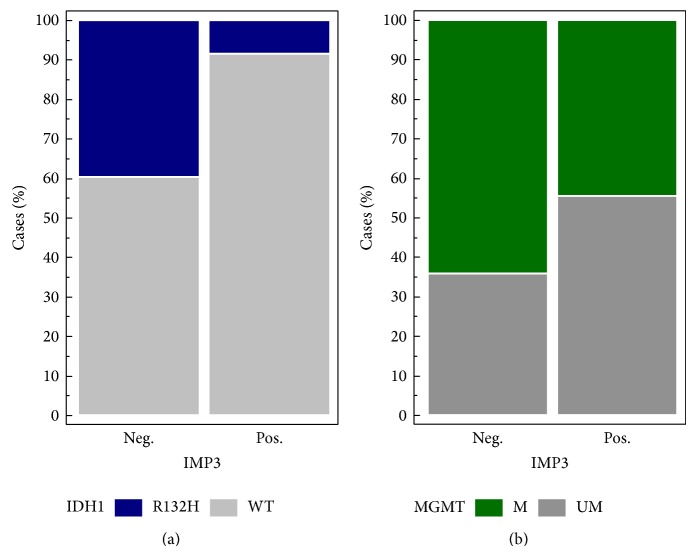
Frequency charts that show association between IDH1 mutation (*P* = 0.0001, (a)) and MGMT methylation status (*P* = 0.004, (b)) with IMP3 protein expression in high-grade gliomas.

IMP3 expression was significantly associated with the absence of mutations of IDH1 gene (*P* = 0.0001) (Figure 4(a)) and with the unmethylated phenotype of MGMT (*P* = 0.004) (Figure 4(b)) in HGG. No statistically significant difference in survival was observed when comparing IMP3- and IDH1-positive high-grade gliomas to IMP3-positive HGG with no IDH1 mutation.

Next we tested whether IMP3 expression was correlated with brain tumor proliferation, assessed by Ki67 antigen expression. In astrocytomas, ultrarapid Ki67 immunostaining was demonstrated to be a useful adjunct to morphological diagnosis and grading, in particular in intraoperative diagnosis of gliomas [8]. Moreover, in anaplastic oligodendroglial tumors, Ki67 index has been demonstrated to have a strong prognostic impact [9].

**Figure 5:**
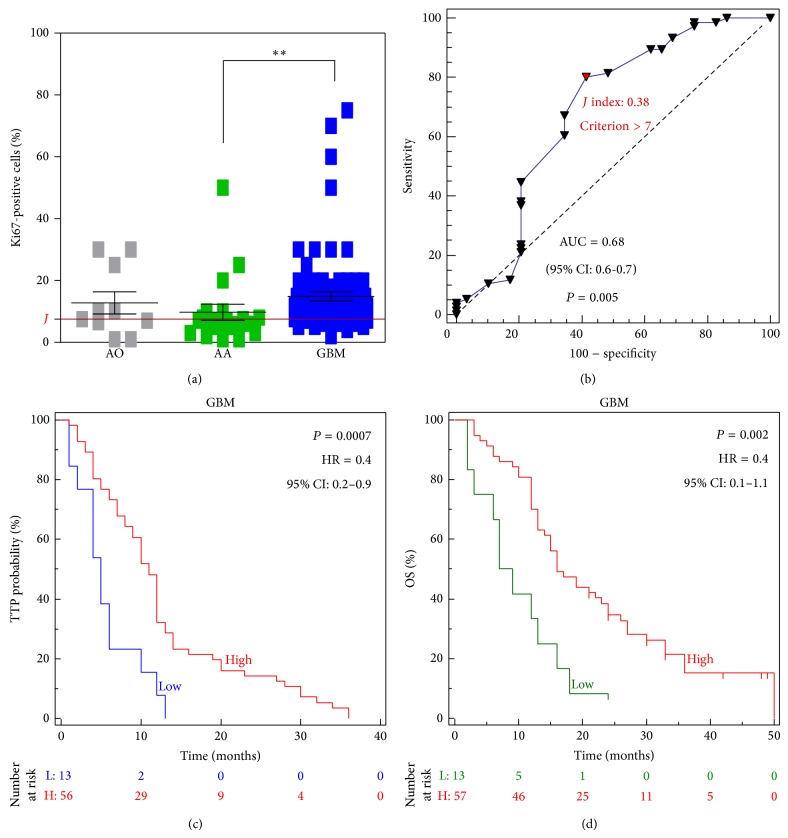
(a) Ki67 labeling index was scored in HGGs as the percentage of positive tumor cells in each case. AO: anaplastic oligodendroglioma; AA: anaplastic astrocytoma; GBM: glioblastoma. ^**^ *P* < 0.01 by *t*-test. (b) ROC curve was used to calculate a cut-off for patients' categorization according to Ki67 levels (*J* index). Disease-free survival (*P* = 0.0007, HR = 0.4, 95% CI: 0.2–0.9) (c) and overall survival (*P* = 0.002, HR = 0.4, 95% CI: 0.1–1.1) (d); end-points were evaluated in GBM patients according to Ki67 index using the Kaplan-Meier method. *P* values are from log-rank test.

Therefore, we correlated IMP3 expression to Ki67 levels detected at diagnosis. Matched data were available for 105 patients, including 29 grade III and 76 grade IV gliomas. GBMs expressed higher Ki67 levels compared to anaplastic astrocytomas (*P* = 0.0014), but IMP3 expression was not correlated with the proliferation index in GBM and grade III tumors. Higher Ki67 levels as determined by ROC curve (*J* index: 0.38, criterion > 7, Figures 5(a) and 5(b)) were correlated with longer DFS (*P* = 0.0007) and OS (*P* = 0.002) in GBM patients (Figures 5(c) and 5(d)).

Finally, we analyzed the survival data in relation to the extent of resection. GBM patients who received a GTR had a better outcome, considering either overall or disease-free survival (*P* < 0.0001 for OS and *P* = 0.0001 for DFS). Conversely, no statistically significant difference was identified for grade III gliomas. We therefore analyzed whether IMP3 expression was an independent prognostic factor. In multivariate analysis, IMP3 lost its prognostic significance, whereas the extent of resection maintained its predictive power (*P* = 0.0009, HR = 0.3, 95% CI: 0.15–0.60 by Cox regression analysis).

## 4. Discussion

In this study, we examined IMP3 protein expression in a series of human gliomas, correlating our results with molecular parameters and Ki67 proliferative index.

IMP3 is a member of the insulin-like growth factor II mRNA-binding protein that regulates mRNA transport, translation, and stabilization, and it is expressed during the early phases of embryogenesis contributing to cell growth and cell migration [1–3].

In particular, in nervous system, IMP3 has been demonstrated in* Xenopus laevis* to be required for neural crest migration, suggesting that this protein is important for promoting cell migration [20].

After birth, IMP3 is epigenetically silenced, with no detectable protein in normal adult tissues.

Several studies demonstrated that IMP3 is overexpressed and plays a role in a wide spectrum of human malignancies and its expression has potential utility in routine surgical pathology practice by discriminating between high-grade preneoplastic lesions and cancer in doubtful cases and providing prognostic information [1–3].

In particular, IMP3 is a useful tool for the diagnosis of gastrointestinal (oesophageal, pancreatic, and biliary), mesothelial, gynaecologic (endometrial serous and squamous cervical), and neuroendocrine carcinomas [4], with very high sensitivity and specificity for these cancers in association with pathological and clinical data [21].

Regarding preinvasive lesions, we recently demonstrated that IMP3 can help in discriminating endometriotic cysts with reactive atypia from cysts lined by preneoplastic atypical endometriosis [5].

As a prognostic marker, different studies demonstrated that IMP3 can identify subgroups of patients affected by localized renal cell carcinomas, superficial urothelial carcinomas, and colorectal carcinomas with poorer prognosis [22].

The only previous study about IMP3 protein expression in adult gliomas focused just on glioblastomas and identified IMP3 as a GBM-specific marker of tumour aggressiveness and of poor prognosis. The authors demonstrated that IMP3 action through IGF-2 results in the activation of oncogenic PI3K and MAPK pathways [15].

Another protein of IMP-family, IMP2, has been demonstrated to regulate oxidative phosphorylation in glioblastomas sphere cultures, and its depletion resulted in impaired clonogenicity and tumorigenicity* in vitro* [23].

In this context, our study, together with* in silico* evidences, demonstrates that IMP3 plays a role in glioma progression and its elevated expression identifies a subset of HGG patients with shorter survival times independently of the tumour grade.

With regard to anaplastic gliomas (grade III), 84% of IMP3-positive cases showed astrocytic differentiation and we could speculate that IMP3 expression is more likely associated with astrocytic lineage.

In relation to molecular characteristics of high-grade gliomas, we correlated IMP3 protein expression with IDH1 mutational and MGMT methylation status.

Epigenetic silencing of the MGMT DNA-repair gene by promoter methylation compromises DNA repair and has been associated with longer survival in patients with glioblastoma who receive alkylating agents and temozolomide [24].

Recent studies confirmed that IDH1 mutation is associated with better prognosis in patients with glioma by inducing cell cycle arrest in G1 phase, inhibiting cell proliferation, and reducing invasion ability, by reducing the levels of matrix metalloproteinases MMP2 and MMP9 [25].

In our study, we investigated IDH1 mutational status through the immunohistochemical antibody mIDH1^R132H^ according to the evidence of a statistically significant correlation between the immunohistochemical staining and the relevant mutation c.395G>A (p.R132H) [26].

Our findings showed that IMP3 protein expression was also significantly associated with IDH1 wild-type phenotype and MGMT unmethylated phenotype in HGG gliomas and we can speculate that the expression of IMP3 in cases without IDH1 mutations and MGMT methylation confirms the role of the former as a negative prognostic marker.

We next studied whether this protein was related to tumor proliferation using Ki67 expression, a nuclear antigen expressed by cells at all cell cycle phases except G0. According to scientific literature and WHO classification, increasing values of Ki67 are correlated with increasing grade of malignancy in human gliomas [8, 9, 27]. Ki67 staining is helpful in differentiating between diffuse astrocytomas and anaplastic astrocytomas, although it cannot discriminate between grade III astrocytoma and GBM. In addition, there is an important overlap of Ki67 values between the different grades of gliomas and there are variations in the proposed cut-off values between different studies. For these reasons, it cannot be used as a diagnostic factor alone but should be used in combination with WHO morphological established criteria [28], or possibly in association with other immunohistochemical markers.

To investigate whether IMP3 could be one of these markers, we looked for a correlation between IMP3 immunohistochemical expression and Ki67 labeling index in the high-grade gliomas. Although both markers were overexpressed in GBM patients compared to grade III gliomas, no significant association between IMP3 and Ki67 could be identified.

In conclusion, this is the first study that investigates the immunohistochemical expression of the IMP3 protein with a correlation with other important parameters (IDH1 mutation and MGMT methylation status) and Ki67 proliferative index in a large series of high-grade gliomas. Our study documents the association between this marker and patients' prognosis in these tumors.

The previous demonstration of IMP3 involvement in PI3K pathway suggests that it could be a target for biological therapies, in particular in combination with PI3K inhibitors, which include more than fifteen drugs that have already progressed in clinical trials [29].

Our results suggest that IMP3 staining could increase the accuracy of histological diagnosis and tumor grading and could identify a subgroup of patients with poor prognosis and at a higher risk of recurrence in high-grade gliomas.

## Figures and Tables

**Table 1:** Clinicopathological characteristics of patients enrolled in the study.

Grade	Age (years)	Gender (M/F)	Ki67 values (%)	MGMT methylated cases	IDH1^R132H^
III (*n* = 29)	49 (22–72)	15/14	7.8 (1–50)	19 (65,5%)	16 (55,2%)
IV (*n* = 106)	55 (24–78)	62/44	10 (2–75)	38 (39%)	11 (11,5%)

**Table 2:** Immunohistochemical results.

Grade	IMP3 (%)	IMP3 score
III (*n* = 29)	6 (20%) 5 AA; 1 AO	4/6 focal (66%) 2/6 diffuse (33%)
IV (*n* = 106)	74 (68%)	31/74 focal (42%) 43/74 diffuse (58%)

**Table 3:** The Oncomine repository^1^ was queried for IMP3 (IGF2BP3) differential expression in brain cancers and only studies performed on human tissues were considered. The name of the study, the samples for which the comparison was significant^2^, the relative expression difference (fold change), and the associated *P* value (*t* statistic provided within the database) are reported.

Study	Comparison^3^	Analysis^4^	*P* value	Fold change
TGCA brain	OD versus normal brain	GEX	1.65*E* − 30	7.03

Nutt brain	GBM versus OD	GEX	1.88*E* − 05	3.2

Sun brain	OD versus normal brain	GEX	1.63*E* − 26	9.07
	OD versus normal brain		1.40*E* − 07	4.04
	AA versus normal brain		6.70*E* − 05	3.2

Murat brain	OD versus normal brain	GEX	1.40*E* − 17	3.67

Liang brain	GBM versus OD and mixed glioma	GEX	7.80*E* − 07	2.2

Bredel brain 2	GBM versus brain cancers	GEX	4.26*E* − 05	2.05
	OD versus normal brain		6.48*E* − 04	4

Shai brain	GBM versus brain cancers	GEX	1.30*E* − 05	1.71
	OD versus normal brain		6.02*E* − 06	1.78

TGCA brain 2	GBM versus brain cancers	CNV	6.07*E* − 53	1.2
	OD versus normal brain		2.40*E* − 08	1.1
	OD versus normal brain		1.20*E* − 150	1.3

Beroukhim brain	OD versus normal brain	CNV	4.30*E* − 11	1.14
	GBM versus brain cancers		5.70*E* − 04	1.08

^1^ https://www.oncomine.org/resource/login.html.

^2^ *P* value less than 1*E* − 4.

^3^GBM: glioblastoma; OD: oligodendroglioma.

^4^GEX: gene expression analysis; CNV: DNA copy number variation analysis.
